# Controlled breeding and reproductive management in water buffaloes (*Bubalus bubalis*) using Eazi Breed controlled internal drug release

**DOI:** 10.4102/jsava.v86i1.1064

**Published:** 2015-06-04

**Authors:** Shivayogi Hiremath, Kerekoppa P. Ramesha

**Affiliations:** 1 Karnataka Co-operative Milk Producers’ Federation Ltd. Training Centre, Dharwad, Karnataka, India; 2 Department of Animal Genetics and Breeding, Indian Council of Agricultural Research – National Dairy Research Institute, Southern Campus, Adugodi, Bengaluru, India

## Abstract

Buffalo reproduction is considerably affected by late maturity, poor oestrus symptoms and long postpartum periods. This study was undertaken to evaluate the efficiency of Eazi Breed controlled internal drug release (CIDR), an intravaginal progesterone-releasing device, in relation to oestrus and fertility. Five hundred true anoestrus buffalo cows, in the age group 4–6 years in 10 villages of Dharwad district in Karnataka state in India, were randomly selected and treated with CIDR for 9 days. Two mL of Cidirol (1 mg oestradiol benzoate) was administered intramuscularly to all animals on day 10. Forty-two buffaloes (8.4%) that failed to show oestrus signs (1.6%) or showed weak signs of oestrus (6.8%) after the first treatment were treated again 72 h after the Cidriol injection with a new device, and inseminated after the expression of oestrus. After the second treatment all the animals showed oestrus signs. The percentage of buffaloes showing intense oestrus was 67.40%, intermediate oestrus was shown by 25.80%, whilst 6.80% buffaloes showed weak oestrus even after the second treatment. The buffaloes showing oestrus signs were inseminated twice with an interval of 12 h, starting 12 h after the start of the oestrus signs. In 86 buffaloes showing prolonged oestrus signs a third insemination was done. The conception rates were 85.16%, 60.47% and 44.11% respectively in buffaloes showing intense, intermediate and weak oestrus. Transrectal palpation of the genital tract was performed 45–60 days post-insemination to diagnose pregnancy status, and in doubtful cases pregnancy was reconfirmed at 90 days after insemination. Out of 500 buffaloes treated in this way 380 animals became pregnant and the pregnancy rate was 76%. This study revealed the usefulness of Eazi Breed CIDR along with Cidirol treatment in buffaloes to improve their reproductive performance.

## Introduction

In India buffaloes contribute to food security through the production of 60 million tons of milk and more than 1 million tons of meat. In addition they provide work energy for agricultural purposes. Buffaloes are found in widely differing geographical areas, which suggests that this species is adaptable to a wide range of environmental conditions.

Besides genetic make-up, several factors such as nutrition, management, environment, physiology, pathology and psychology affect the conception rate in buffaloes under both farm and field conditions, and pose a serious threat to profitable farming. The profitability of buffalo farming mainly depends upon their regular and efficient breeding. Reproductive efficiency of buffaloes is considerably affected by late maturity, poor oestrus signs and long postpartum intervals.

Anoestrus due to ovarian inactivity is considered to be the most important cause of lowered fertility in buffaloes, and is responsible for tremendous economic losses to farmers by decreasing milk yields in addition to lowering calf production. True anoestrus is a condition in which both ovaries are small, smooth, inactive with the absence of a Graafian follicle or corpus luteum, and characterised by cessation of the sexual cycle and psychic manifestation of oestrus (Nayak *et al*. 2009). The incidence of true anoestrus (Tanwar, Rakha & Phogat 2003) and silent oestrus or suboestrus on the Indian subcontinent is higher in buffaloes (Shah, Willemse & Vandewiel 1990) than in cattle (Yadav *et al*. 2004). With this background, the present study was undertaken on anoestrus buffaloes to evaluate the efficiency of the Eazi Breed controlled internal drug release (CIDR) intravaginal progesterone-releasing device and oestradiol administration in relation to oestrus and fertility.

An increased calving-to-conception interval as a result of true anoestrus or suboestrus in bovines adversely affects the economics of the dairy sector. Several factors, such as breed, parity, season (Singh 2001), climate, temperature, photoperiod, nutrition, age, body weight, level of production, hormonal imbalance (Suthar & Dhami 2010), presence of a bull, negative energy balance (Butler & Smith 1980) and suckling (Stagg *et al*. 1998), were indicated as affecting the length of the oestrus cycle and the degree of heat expression. Oestrus behaviour in buffalo cows has a lower intensity than in domestic cows, and is therefore much more difficult to detect. The buffalo cow in oestrus is rarely observed to mount other buffaloes or stand to be mounted by other females (Chaudhry, Ahmad & Khan 1988). Signs such as swollen vulva, mucous discharge from the vulva and increased frequency of urination are not regarded as reliable indicators of oestrus (Luktuke & Roy 1964). Moreover, the frequency of oestrus activity is reduced during day time (Usmani, Ahmad & Inskeep 1984). Therefore, before adopting any treatment regimens critical differential diagnosis of true anoestrus and suboestrus is essential in buffaloes.

Use of equine chorionic gonadotrophin (eCG) after a period of progesterone treatment to stimulate cyclicity has been attempted with variable success (Galloway *et al*. 1987; Singh *et al*. 1988). A combination of CIDR for 7 days followed by administration of 1 mg oestradiol benzoate (Lammoglia *et al*. 1998) were also tried in cows. Treatment of bovines suffering from suboestrus has mainly focused on the use of gonadotrophin-releasing hormone (GnRH) and PGF2α (Plunkett, Stevenson & Call 1984; Stevenson *et al*. 2000). Providing anoestrus cows with a short period (5–9 days) of exposure to a progestin can influence many of the components of a successful oestrus-induction programme (Day 2004), and is the central treatment to induce resumption of oestrus cycles.

Totewad *et al*. (2009) reported induction of oestrus using cloprostenol by the intravulvo-submucosal route in suboestrus buffaloes. Patel *et al*. (2003) reported induction of oestrus in buffaloes using norgestomet ear implants. Different hormonal treatments, like oestrogen, progesterone and GnRH alone or in combination, have been tried with variable success for treatment of anoestrus cattle and buffaloes (Rao & Rao 1984; Rao, Srimanarayana & Rao 1985; Singh, Saxena & Prasad 2004).

Against this background the present study was undertaken on true anoestrus buffaloes to study the efficiency of an Eazi Breed CIDR intravaginal progesterone-releasing device and Cidirol treatment in relation to oestrus and fertility.

## Research method and design

The study was carried out in 10 different villages in the Dharwad milkshed area of Karnataka, India. Dharwad district is situated in the western sector of the northern part of Karnataka state in India. The district encompasses an area of 4263 km^2^, lying between the latitudinal parallels of 15^°^02’ N and 15^°^51’ N and longitudes of 73^°^43’ E and 75^°^35’ E. Dharwad is located at a mean height of 671 m.a.s.l. Annual rainfall in the experimental areas of Dharwad district ranges from 80 cm to 95 cm and temperature ranges from 12 °C to 39 °C, with the rainy season from June to September.

A baseline survey was conducted and nutritional and reproductive conditions of each buffalo cow were examined and recorded. The buffaloes that failed to show oestrus signs for 8–10 months after calving were examined per rectum, and buffalo cows with inactive ovaries and no cyclic activity were diagnosed to be in true anoestrus. Five hundred anoestrus buffaloes aged between 4 and 6 years, belonging to 10 different villages with 45–55 buffaloes from each village, were selected randomly and included as experimental animals in the study. An Eazi Breed CIDR (1.9 g natural progesterone) device was inserted into these animals using the specially designed applicator in accordance with the directions of the manufacturers. The device remained in the vaginal cavity for 9 days. In 5% of the buffalo cows the device was lost and a second new Eazi Breed CIDR device was inserted within 24 h.

The intravaginal progesterone-releasing devices deliver progesterone at a controlled rate into the bloodstream of the animals. The progesterone is released by diffusion from artificial silicone rubber moulded over a nylon spine shaped to retain the device in the vaginal cavity. Removal of the device at the end of the treatment period was easily achieved by gentle but firm pulling on the tail of the device. Oestrus was detected by the critical observation of behaviour and later confirmed by rectal examination of the animals. Twenty-four hours after removal of the device, all animals were treated with 2 mL Cidirol (1 mg estradiol benzoate) intramuscularly, and more pronounced external oestrus signs were observed.

The buffaloes were inseminated 48 h (range 24–72 h) after withdrawal of the device. The buffalo cows showing oestrus signs were inseminated twice with an interval of 12 h, beginning at 12 h after the start of the oestrus signs. In 86 buffalo cows showing prolonged oestrus symptoms a third insemination was done using fresh semen brought from Karnataka Co-operative Milk Producers’ Federation Ltd. (KMF) Nandini sperm station at Hessaraghatta, Bangalore. Oestrus intensity (standing, discharge and mucous flow) was recorded as intense, intermediate or weak.

All of the inseminated buffaloes were examined per rectum on the 12th and 15th day post-artificial insemination to detect the presence of a corpus luteum. Pregnancy was confirmed by transrectal examination of the genital tract on the 45th–60th day after artificial insemination. Doubtful cases were re-examined on the 90th day. The animals that failed to show oestrus (1.6%) as well as those that showed weak signs of oestrus (6.8%) after first treatment were treated again 72 h after the Cidriol injection by inserting a new device, and inseminated after the expression of oestrus.

The conception rate was calculated by dividing the number of pregnant buffaloes by the total number of buffaloes inseminated. Pearson chi-square analysis was applied to test the significance of different oestrus signs and conception rates and how these related to different villages.

## Results

After removal of the Eazi Breed CIDR device implants, anoestrus buffalo cows showed different external signs of oestrus. The majority of the animals showed hyperaemia of the vaginal mucosa and frequent urination. The percentage of animals on heat did not change with the injection of Cidirol, but external heat signs after injection of 2 mL of Cidirol were more intense. The percentage of animals showing different external oestrus signs are presented in Table 1. All the buffalo cows with weak oestrus signs (6.8%) and no oestrus signs (1.6%) that were treated for a second time showed oestrus signs after the second treatment. The conception rate amongst the 42 buffaloes that received a second treatment was 42.86%.The conception rate after second treatment and artificial insemination amongst those buffalo cows that failed to show oestrus signs after the first treatment was 37.5%.

**TABLE 1:** Percentage of animals showing the particular signs of oestrus.

Signs	Animals showing symptoms (%)	SE (%)
Hyperaemia of vagina	86	1.55
Frequent urination	83	1.68
Free-flowing mucous	76	1.91
Bellowing	68	2.09
Raised tail	63	2.16
Excitement or restlessness	62	2.17
Reduced feed and water intake	50	2.24
Low milk yield	40	2.19
Swollen vulva	38	2.17
Allowing mounting	20	1.79
Licking other animals	12	1.45
Mounting other animals	10	1.34

SE, standard error.

Upon the removal of the device, all of the animals that responded to the treatment exhibited oestrus signs within 24–72 h, with the duration ranging from 36 to 48 h. The percentage of buffaloes showing intense oestrus was 67.40%, while intermediate oestrus was shown by 25.80% and weak oestrus was observed in 6.80% (Table 2). The corresponding conception rates were 85.16%, 60.47% and 44.11% respectively in buffalo cows showing intense, intermediate and weak oestrus (Table 2).

**TABLE 2:** Conception rates in 10 villages amongst buffaloes showing intense, intermediate and weak oestrus signs.

Village number	Name of village	Number of animals	Intense signs				Intermediate signs				Weak signs			
			Animals showing oestrus symptoms		Animals that conceived		Animals showing oestrus symptoms		Animals that conceived		Animals showing oestrus symptoms		Animals that conceived	
			*n*	*%*	*n*	*%*	*n*	*%*	*n*	*%*	*n*	*%*	*n*	*%*
1	Byhatti	52	37	71.2	28	75.7	13	25.0	7	53.9	2	3.8	1	50.0
2	Hebbasur	46	28	60.9	23	82.1	16	34.8	8	50.0	2	4.3	1	50.0
3	Kusgal	48	30	62.5	25	83.3	15	31.2	8	53.3	3	6.2	1	33.3
4	Lokur	45	32	71.1	27	84.3	12	26.7	7	58.4	1	2.2	0	0.0
5	Mugali	53	34	64.2	30	88.2	15	28.3	8	53.3	4	7.5	2	50.0
6	Madhanbhavi	56	36	64.3	30	83.3	16	28.6	12	75.0	4	7.1	1	25.0
7	Padesur	54	38	70.4	32	84.2	12	22.2	10	83.3	4	7.4	2	50.0
8	Shirol	54	38	70.4	34	89.4	10	18.5	6	60.0	6	11.1	3	50.0
9	Yadawad	45	32	71.1	28	87.5	11	24.4	7	63.7	2	4.4	1	50.0
10	Yamanoor	47	32	68.1	30	93.7	9	19.1	5	55.5	6	12.8	3	50.0
**Total**	-	**500**	**337**	**67.40**	**287**	**85.16**	**129**	**25.80**	**78**	**60.47**	**34**	**6.80**	**15**	**44.11**

Pearson chi-square analysis indicated that development of the signs of oestrus was independent of villages (*χ*2 = 12.50, *p* > 0.05) and also that the conception rates amongst the villages were similar (*χ*2 = 5.37, *p* > 0.05). The conception rate was high in buffaloes exhibiting intense oestrus signs, followed by animals showing intermediate signs, and least in animals showing weak oestrus signs (*χ*2 = 51.53, *p* > 0.001).

## Discussion

The number of buffaloes showing intense heat (67.40%) and high conception rates after the Eazi Breed CIDR along with Cidirol treatment is higher in this study compared with the earlier reports of Chede (1990) and Nayak *et al*. (2009). After CIDR implant use, Shrivastava and Kharche (1985) reported intense, intermediate and weak oestrus signs in 27%, 32% and 41% of the buffaloes respectively, which is lower than the results of the present study. The better oestrus signs and fertility obtained in this study could be attributed to the Eazi Breed CIDR cattle device and Cidirol injection, which stimulated follicular development and ovulation, and also to insemination twice or three times using fresh semen. The variation could also be due to the nutritional and health status of the buffalo cows under study.

The use of CIDR ensures that animals receive the necessary dose of progesterone to inhibit ovulation during the presence of the device. The buffalo cows were in anoestrus, hence no corpus luteum was present at the start of the experiment. The CIDR implant ensures controlled release of progesterone over a 9-day period. After 9 days the insert is removed. Removal of the CIDR device causes a decrease in blood levels of progesterone. This allows growth of the dominant follicle and leads to an increase in blood concentrations of oestradiol, which leads to the expression of oestrus signs. Intramuscular injection of oestradiol benzoate 24 h after removal of the CIDR device leads to a further increase in blood oestradiol concentrations, with an increase in the intensity of oestrus signs. Increased blood oestradiol concentration has a positive feedback on the GnRH pulse regulator mechanism, which in turn induces the luteinising hormone surge. High GnRH pulse frequency causes the pituitary to release luteinising hormone.

Srivastava (2005) reported that conception in buffaloes was not very encouraging (50%) after artificial insemination, but oestrus was induced in 75% of the buffaloes after administering 100 mg hydroxy progesterone intramuscularly twice daily for 8 days. Similarly, Abdoon, Ahmed and Tochamy (1994) observed only 40% of buffalo heifers in oestrus when oestradiol and progesterone treatment was used for oestrus induction. Luthra, Khar and Singh (1994) reported an 86% oestrus induction response and 57% conception rate after artificial insemination in buffaloes following use of a progestagen implant, which is lower than the results obtained in this study.

Kamimura *et al*. (2000) and Abdullah *et al*. (2001) also found 86% and 100% induced oestrus respectively using long-term progesterone (CIDR) in crossbred cows. Anwer *et al*. (2003) also induced oestrus in 71% and 100% of buffaloes using GnRH and cloprostenol treatment amongst true anoestrus animals in rural and peri-urban areas respectively, and 100% in silent oestrus buffaloes.

The results of this study strongly suggest that use of the Eazi Breed CIDR device along with Cidirol treatment is beneficial in buffaloes diagnosed with true anoestrus and in buffaloes with poor oestrus symptoms. After treatment of true anoestrus buffalo cows with the Eazi Breed CIDR device along with Cidirol treatment, the conception rate was higher than in earlier reports in buffaloes. Signs of oestrus and conception rates were independent of the villages, further strengthening evidence of the usefulness of the treatment.

This study revealed the usefulness of Eazi Breed CIDR along with Cidirol treatment in buffaloes to improve reproductive performance. This could be one of the possible ways to improve reproductive performance in buffaloes, in addition to better feeding and management.

## Conclusion

The Eazi Breed CIDR device along with Cidirol treatment is beneficial to bring anoestrus buffaloes into oestrus and to improve reproductive performance in these animals. This technique could be used to enhance reproductive performance in water buffaloes.
